# Sucrose-Based Macrocycles: An Update

**DOI:** 10.3390/molecules30132721

**Published:** 2025-06-24

**Authors:** Sławomir Jarosz, Zbigniew Pakulski

**Affiliations:** Institute of Organic Chemistry, Polish Academy of Sciences, Kasprzaka 44/52, 01-224 Warsaw, Poland; zbigniew.pakulski@icho.edu.pl

**Keywords:** sucrose in fine organic synthesis, macrocyclic derivatives with sucrose scaffold, molecular cages based on sucrose and cycloveratrylene, complexing properties of macrocycles with sucrose scaffold, sucrose-based molecular switches

## Abstract

Sucrose is by far the most abundant disaccharide found in nature, consisting of two simple hexose units: d-glucose and d-fructose. This exceptionally inexpensive and widely accessible raw material is produced in virtually limitless quantities. The vast majority is consumed in the food industry either in its native form—as commercial table sugar—or, to a lesser extent, as the basis for artificial sweeteners such as palatinose and sucralose. Beyond its dietary use, sucrose serves as a feedstock for the production of bioethanol, liquid crystals, biodegradable surfactants, and polymers. However, the application of this valuable and extremely cheap raw material (100% optical purity and eight stereogenic centers with precisely defined stereochemistry) in the synthesis of more sophisticated products remains surprisingly limited. In this short review, we focus on the strategic use of the sucrose scaffold in the design and synthesis of fine chemicals. Special attention will be paid to macrocyclic derivatives incorporating the sucrose backbone. These water-soluble structures show promise as molecular receptors within biological environments, offering unique advantages in terms of solubility, biocompatibility, and stereochemical precision.

## 1. Introduction

**Figure 1:**
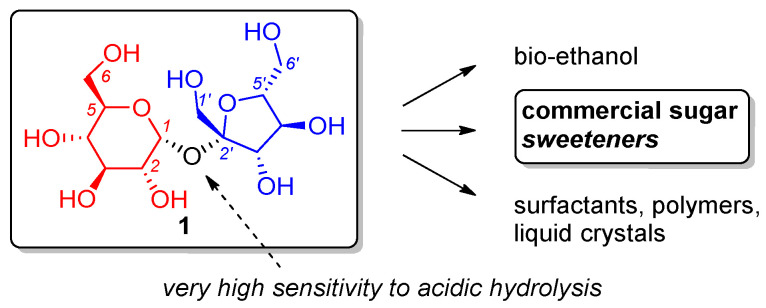
Sucrose [**1**; α-d-glucopyranosyl-β-d-fructofuranoside]: its presence in food chemistry and as a starting material for industrial biodegradable products.

Sucrose (**1**; α-d-glucopyranosyl-β-d-fructofuranoside), a very cheap raw material produced in plants (sugar cane and sugar beets) from the primary products of photosynthesis, is available in practically unlimited quantities. The majority of commercially available sucrose is consumed in its native form as table sugar, primarily in the food industry. To a lesser extent, it also serves as a precursor for the industrial-scale production of sucrose-derived sweeteners such as palatinose and sucralose. Beyond its applications in food technology, sucrose plays an increasingly important role in industrial chemistry. One of its major non-food uses is in the production of bioethanol, where it serves as a fermentable sugar source. Moreover, sucrose has emerged as a versatile starting material in the synthesis of high-value compounds, including liquid crystals, biodegradable surfactants, polymers, and, potentially, cytotoxic antitumor agents (Figure 1) [1].

Despite its exceptional advantages—such as 100% optical purity and eight stereogenic centers with precisely defined stereochemistry—the utilization of sucrose in the synthesis of complex fine chemicals remains surprisingly underexplored. The potential of this structurally rich and sustainable molecule for advanced chemical synthesis has only been modestly realized. Some of these synthetic applications have been discussed in our recent reviews [2,3].

**Figure 2:**
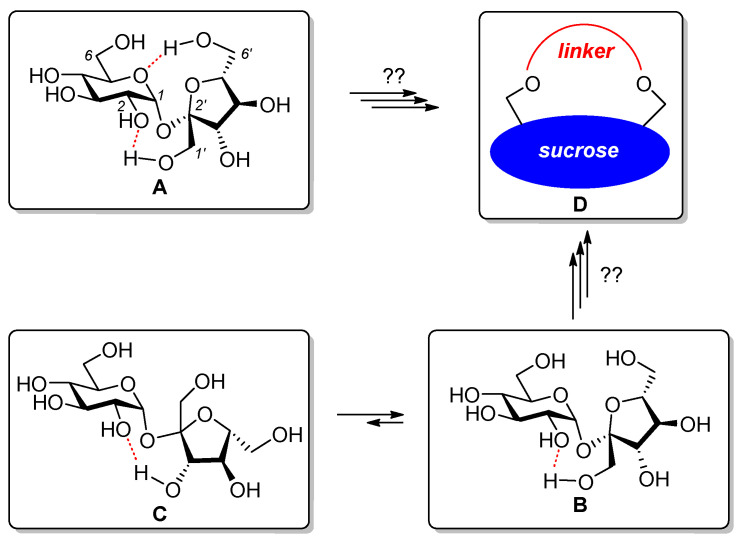
Conformation of sucrose in solid state (**A**) and in solution (**B**,**C**). Possible macrocyclic target with sucrose scaffold (**D**).

The conformation of sucrose in both the solid state and in solution indicated that the primary hydroxyl groups at the C6 position of the glucose moiety and the C6′ position of the fructose moiety are in close spatial proximity due to strong intramolecular hydrogen bonding, which suggests the feasibility of covalently linking these two positions via a suitable linker (Figure 2) [4].

**Figure 3:**
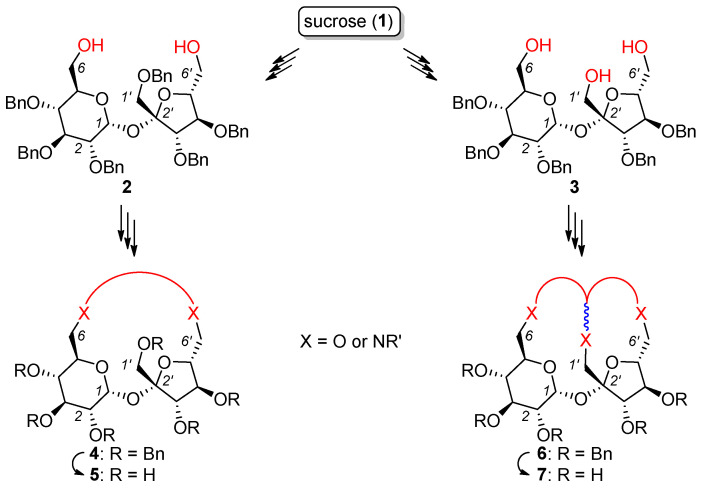
Synthetic plan for the preparation of soluble sucrose-based receptors.

Such a possibility may open a route to highly functionalized macrocyclic derivatives that are soluble in water, which would allow these compounds to be used as receptors in biological environments. The attempt to utilize sucrose-based macrocycles requires several steps (Figure 3):

- The protection of all secondary hydroxyl groups with temporary blocks (preferably benzyl) that could be easily removed after the desired transformations without the destruction of a highly sensitive glycosidic bond (Figure 3; compounds **2** and **3**). This strategy requires temporary protection of the primary hydroxyl groups (C6, C6′ and eventually C1′), benzylation of all remaining OH groups, and the removal of temporary blocks from C6, C6′ (and eventually C1′) positions. The selective protection of the primary OH groups is well known and can be achieved using very expensive bulky silyl chlorides or much cheaper trityl chlorides. However, the removal of the trityl blocks from per-benzylated intermediates was not trivial since this process can ***only*** be realized by careful acidic hydrolysis.
- The connection of the terminal positions (C6, C6′ and eventually C1′) should be connected via a proper link (**4** and **6**).
- Total deprotection, i.e., the removal of all temporary blocks, which should provide macrocyclic derivatives (potential receptors) soluble in water (**5** and **7**).

**Scheme 1:**
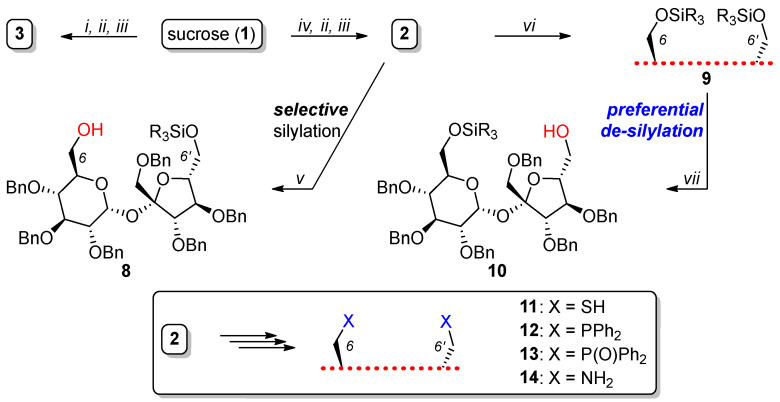
Synthesis of sucrose diol **2**, sucrose triol **3**, and their partially protected derivatives **11–14**. Reagents and conditions: *i*. 3 equiv. TrCl; *ii*. BnBr, NaH; *iii*. careful detritylation; *iv*. 6 equiv. TrCl; *v*. 1 equiv. R_3_SiCl; *vi*. excess of R_3_SiCl; *vii*. F^(-)^.

During our work on the application of sucrose as a valuable synthon for macrocyclic, water-soluble derivatives, we describe a convenient methodology for the synthesis of so-called “sucrose diol” (**2**) and “sucrose triol” (**3**) and other useful partially protected derivatives of this disaccharide; examples are shown in Scheme 1.

Selective silylation of diol **2** afforded product **8** with the free C6-OH group with a very small amount of double-protected compound **9**. This high selectivity was rather expected in view of the early observation by Khan, who reported that the reaction of free sucrose with *tert*-BDPS-Cl afforded 49% of the mono-silylated product in which the silyl group was located at the C6′ (fructose part) position [5].

The double silylated compound **9** could be obtained in high yield by the exhaustive silylation of diol **2**. The most important (not expected) observation in our study was the ***selective de-silylation*** of **9,** which opened a convenient route to regioisomeric mono-alcohol **10** with the free C6′-OH (fructose part) group (Scheme 1). The sulfur, amino-, and phosphorus derivatives were also available, as shown in Scheme 1 [2,3].

## 2. Sugar Derived Macrocyclic Derivatives with Sucrose Scaffold

The best-known macrocyclic derivatives with sugar platform are—undoubtedly—cyclodextrins, which were obtained from the enzymatic degradation of starch by the end of the XIXth century by French scientist Antoine Villiers. In the second half of the XXth century, the method for their industrial preparation was elaborated by Hungarian chemists. These cyclic oligosaccharides have found widespread applications in chemistry, medicine, and daily life [6,7]. Other macrocyclic sugar-based derivatives are also known [8]. We decided to use sucrose—the most common disaccharide—as a platform to build macrocyclic derivatives, which, being soluble in water, could act as a receptor in a biological environment.

### 2.1. Synthesis of Macrocyclic Derivatives from “Sucrose Diol” ***2***

**Scheme 2:**
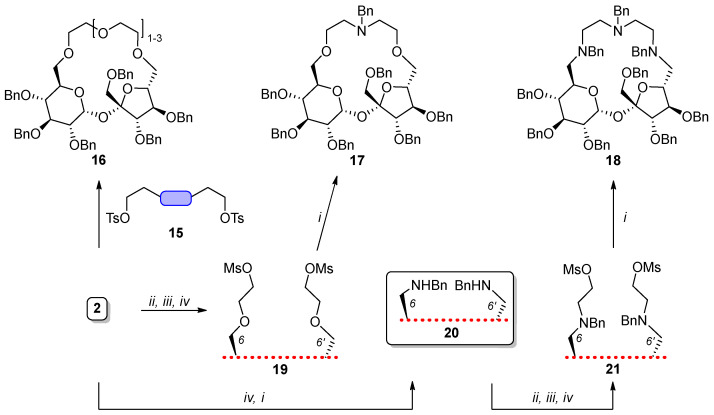
Synthesis of aza-crown ether analogs with sucrose scaffold from diol **2**. Reagents and conditions: *i*. BnNH_2_; *ii*. BrCH_2_CO_2_R; *iii*. LiAlH_4_; *iv*. MsCl.

Diol **2** served as a starting material for the preparation of the analogs of crown and aza-crown ethers. Several such targets built on a sucrose platform were prepared by our group; model examples are shown in Scheme 2 [9,10].

For example, diol **2** was treated with polyethylene glycol di-tosylates (**15**), affording crown ether analog **16**. The preparation of aza-crowns required slightly different options. Thus, the elongation of **2** by two carbon atoms at each side (→ **19**) followed by a reaction with benzylamine provided macrocycle **17** with one nitrogen atom in the ring. The synthesis of macrocycles with more nitrogen atoms was also possible and was exemplified by compound **18**. It was prepared in a few steps from diol **2** by its conversion into di-amine **20,** which was elongated to **21** (similarly to **19**) and finally treated with benzylamine, affording **18**.

**Scheme 3:**
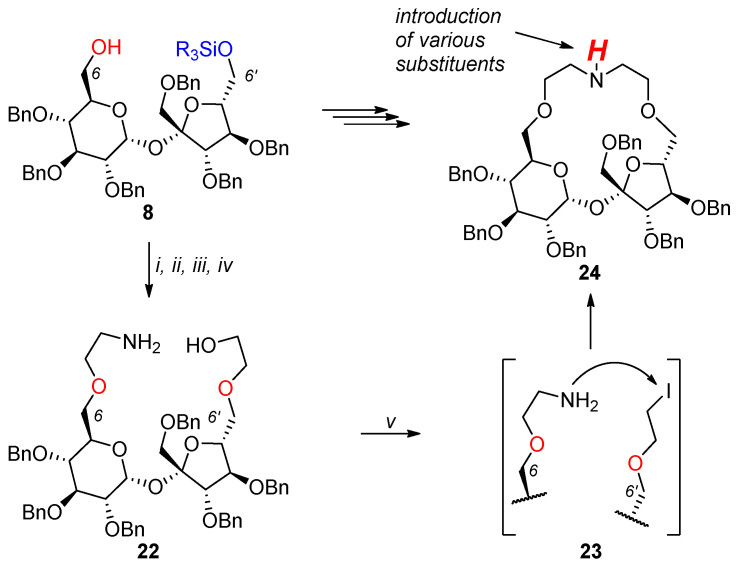
Efficient synthesis of macrocyclic sucrose template. Reagents and conditions: *i*. ClCH_2_CN; *ii*. F^(-)^; *iii*. BrCH_2_CO_2_^t^Bu; *iv*. LiAlH_4_; *v*. imidazole, PPh_3_, I_2_.

In aza-macrocycles **17** and **18,** the nitrogen atom in the ring is protected with the benzyl group; it would have been much more convenient to have this atom free, which would have allowed us to introduce various substituents modifying the properties of the target and such a possibility is shown in Scheme 3 [11]. Starting from alcohol **8,** it was possible to introduce different units at the position C6 (via a reaction with chloroacetonitrile) and C6′ (via a reaction with *^t^*butyl bromoacetate after the deprotection of 6′-OH); this intermediate was subsequently reduced to aminoalcohol **22**. This compound was converted, under Garegg’s conditions (imidazole, I_2_, Ph_3_P), to iodoamine **23,** which spontaneously cyclized to aza-crown **24**. The alkylation of the nitrogen atom afforded a series of crown ether analogs with a sucrose scaffold [12].

**Scheme 4:**
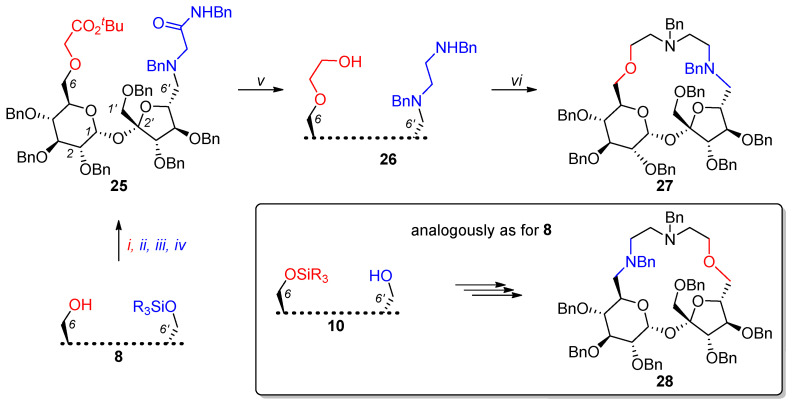
Synthesis of “unsymmetrical” analogs of aza-crowns with sucrose scaffold. Reagents and conditions: *i*. BrCH_2_CO_2_^t^Bu; *ii*. F^(-)^; *iii*. Swern oxidation; *iv*. BnNHCH_2_C(O)NHBn, NaBH_3_CN; *v*. LiAlH_4_; *vi*. imidazole, PPh_3_, I_2_.

The macrocyclic derivatives presented in Scheme 2 and Scheme 3 are “symmetrical”, i.e., they have the same atoms (oxygen or nitrogen) at both “ends” of sucrose (positions C6 and C6′). The synthesis of “unsymmetrical” aza-crown analogs was, however, possible, as shown in Scheme 4. Alcohol **8** was converted into **25** and further into aminoalcohol **26**. The cyclization of this intermediate, induced by a Garegg’s reagent, provided the first “unsymmetrical” azacrown analog **27**. The second macrocyclic analog **28** was prepared analogously from regioisomeric alcohol **10** [12].

**Scheme 5:**
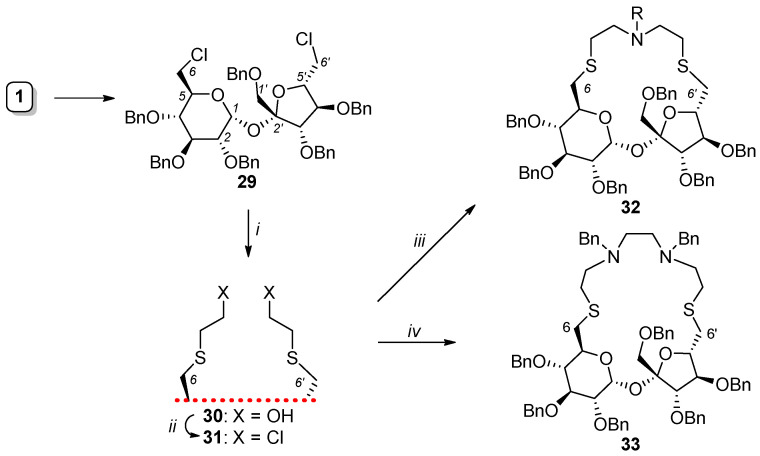
Efficient preparation of sucrose macrocycles with two sulfur atoms. Reagents and conditions: *i*. NaI, 2-mercaptoethanol, DMF; *ii*. CCl_4_, PPh_3_; *iii*. RNH_2_; *iv*. BnNHCH_2_CH_2_NHBn.

We also elaborated on the convenient method for the synthesis of macrocyclic sucrose-based derivatives containing sulfur, which is shown in Scheme 5. Sucrose (**1**) was selectively chlorinated according to the Whistler procedure [13]; then, it was perbenzylated to **29** [14]. The reaction of this intermediate with 2-mercaptoethanol provided diol **30** (X = OH), which was chlorinated (to **31**; X = Cl) under Appel conditions. The treatment of this dichloride with either amines (e.g., BnNH_2_) or di-amines (BnNHCH_2_CH_2_NHBn) afforded macrocyclic derivatives **32** or **33**, respectively, with a sucrose scaffold [15].

### 2.2. Synthesis of Other Complex Macrocyclic Sucrose-Based Derivatives

**Scheme 6:**
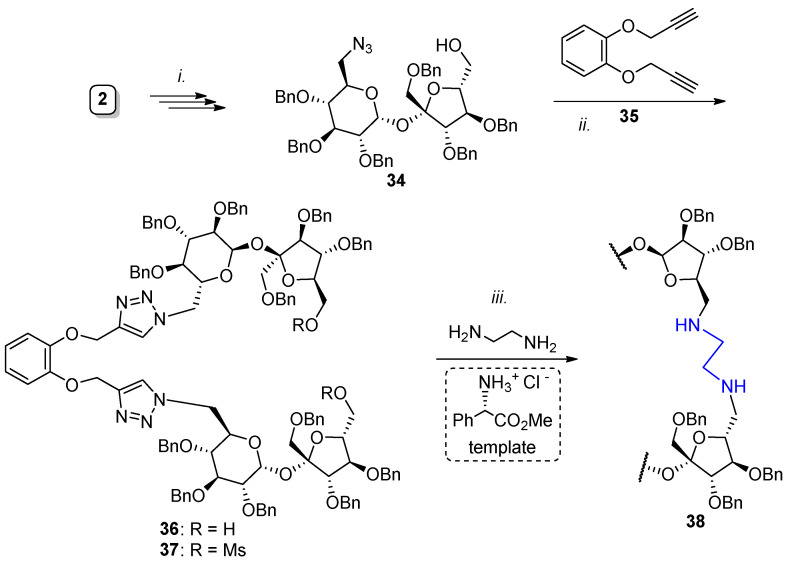
The synthesis of complex macrocyclic derivative **38**. *i.* Ref. [9]; *ii*. CuSO_4_, Na-ascorbate, *tert-*BuOH/H_2_O, 80% and then, MsCl, Et_3_N, DMAP, 95%; *iii.* Na_2_CO_3_, MeCN, reflux, 48h: 5% without a template and 25% with a template.

One of the first sucrose-based macrocyclic derivatives—that was not the crown analog—was prepared by us in 2010, as shown in Scheme 6 [16]. Sucrose diol **2** was converted using a few well-defined steps into azido-alcohol **34,** which—upon reaction with di-acetylene **35**—provided precursor **36** (R = H) at an 80% yield. The activation of both hydroxyl groups as mesylates (**37**; R = Ms), followed by their reaction with ethylenediamine, afforded macrocycle **38** at a 5% yield. However, when this process was carried out in the presence of a template (l-phenylglycine methyl ester hydrochloride), this yield increased to 25%.

**Scheme 7:**
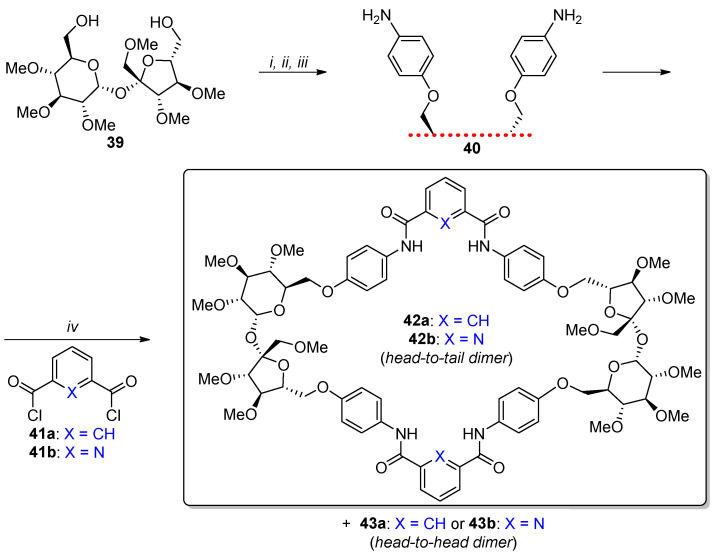
The synthesis of sucrose dimers from **39**. Reagents and conditions: *i*. MsCl, Et_3_N, 90%; *ii*. *p*-nitrophenol, K_2_CO_3_; *iii*. H_2_, 10%Pd/C; *iv*. Et_3_N, **42a** + **43a** (62%) or **42b** + **43b** (54%); both dimers HT and HH were formed at a ratio of 1:1.

Soon after this pioneering work, we introduced a method for the synthesis of other dimers with a sucrose scaffold, as shown in Scheme 7. Diol **39** (permethylated analog of **2**) was converted after mesylation into di-amino derivative **40,** which—upon reaction either with di-acid chloride **41a** or **41b**—gave dimer **42** (head-to-tail dimer a or b) together with its head-to-head analog **43** [17].

**Scheme 8:**
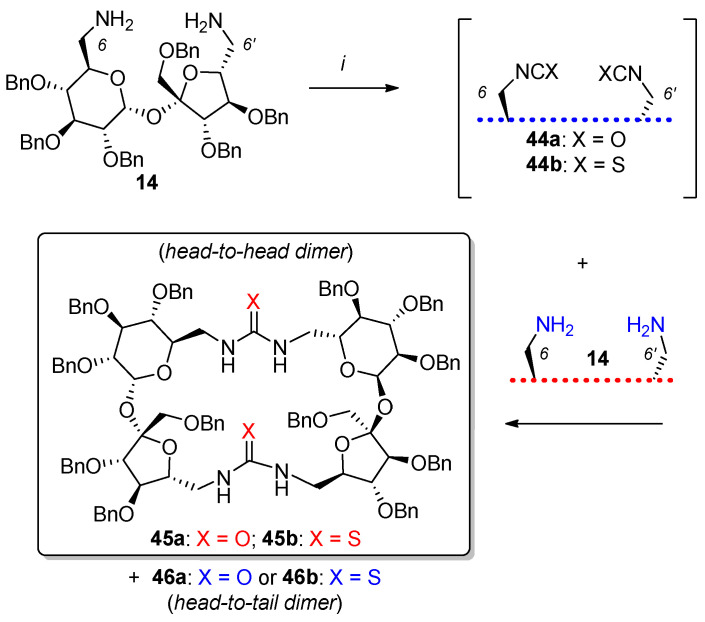
Synthesis of urea and thiourea sucrose dimers. Reagents and conditions: *i*. triphosgene → **44a** or thiophosgene → **44b;** then, **14**, THF, Et_3_N, −10 °C high dilution (90% for **45a** + **46a**; ref. [18]) or Na_2_CO_3_, AcOEt/H_2_O, rt (84% for **45b** + **46b**; ref. [19]).

The synthesis of urea- and thiourea sucrose dimers is shown in Scheme 8 [18,19]. The treatment of diamine **14** with triphosgene or thiophosgene afforded intermediates **44a** or **44b**, which were further reacted with diamine **14** to afford urea and thiourea derivatives **45** (head-to-head) and **46** (head-to-tail) dimers. These macrocycles show high affinity to chloride and acetate anions.

### 2.3. Synthesis of Macrocyclic Derivatives from “Sucrose Triol” ***3***

**Scheme 9:**
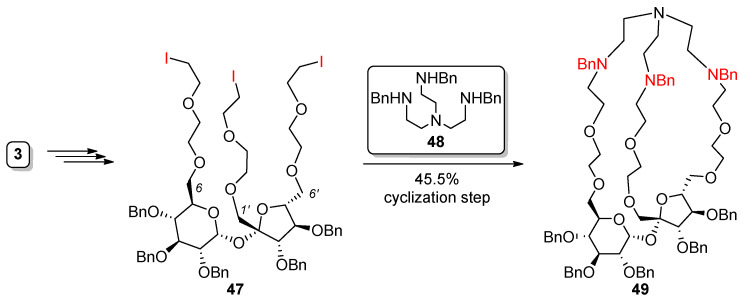
First synthesis of sucrose-based cryptand.

After the successful preparation of the relatively simple analogs of crown and aza-crown ethers (some of which showed interesting complexing properties; see the next chapter), we turned our attention to more sterically demanding molecules in which the molecular cavity responsible for complexation would be much more rigid (see models **6** and/or **7** in Figure 3). Such a possibility requires the connection of all three “ends” of the sucrose molecule, i.e., positions C1′, C6, and C6′. The first example of such a complex derivative, shown in Scheme 9, was prepared by us in 2019 [20]. Triol **3** was used as the starting material for the preparation of sucrose-based cryptands; this was the first (and up-to-date the only one) example of such derivatives. The elongation of all three terminal positions of triol **3** by a –CH_2_CH_2_-O-CH_2_CH_2_-I unit (→ **47**) followed by a reaction with tripodal amine **48** afforded cryptand **49** at a very high yield (45.5%).

**Scheme 10:**
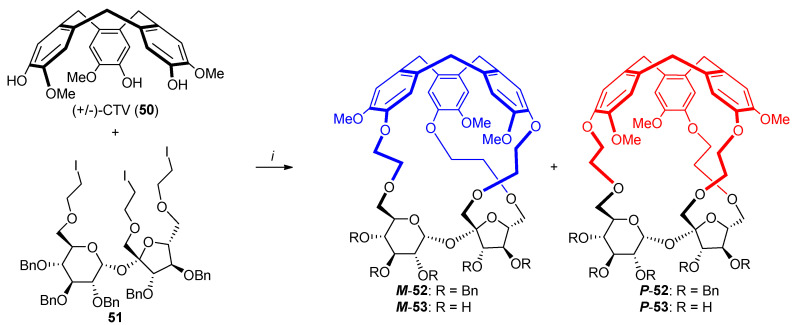
Synthesis of molecular cages **52** and **53** from elongated sucrose triiodide **51** and racemic CTV (**50**). Reagents and conditions: *i*. Cs_2_CO_3_, acetonitrile, and reflux.

A special case is represented, however, by the objects in which the sucrose platform is connected to a tripodal ***chiral*** unit. Thus, the reaction of a *racemic* C_3_-symmetrical cyclotriveratrylene unit (CTV, **50**) with elongated (by two carbon atoms at all terminal positions) sucrose triiodide **51** provided two (out of four possible) diastereoisomeric cages, ***M*-52** and ***P*-52,** as shown in Scheme 10. The benzyl groups protecting the sucrose backbone were easily removed by simple hydrogenation-affording capsules that were soluble in water (***M*-53** and ***P*-53**) [21]. The formation of only two diastereoisomeric cages (**52**) from **51** and ***rac*-50** resulted (most likely) from the fact that the ethylene linker connecting both units was too short.

**Scheme 11:**
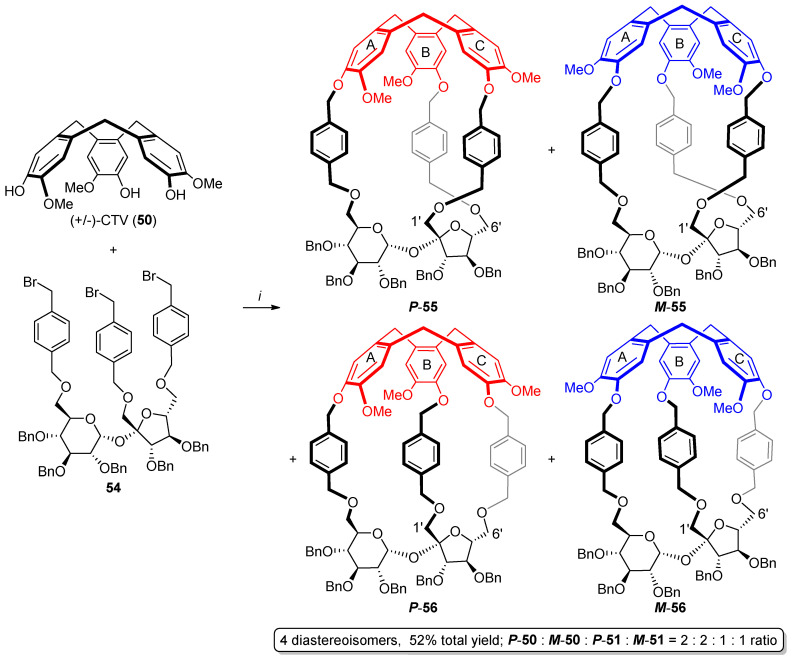
The formation of all four possible distereoisomeric cages in the reaction of *rac*-CTV (**50**) with a “longer” analog **54**. Reagents and conditions: *i*. Cs_2_CO_3_, acetonitrile, and reflux.

For longer than the ethylene linker, the possibility of the formation of all four possible stereoisomers increased significantly. Indeed, the reaction of compound **54** (another analog of sucrose triol **3**) with racemic CTV (**50**) afforded four diastereoisomeric cages (***P*-55**, ***M*-55**, ***P*-56**, and ***M*-56**), as shown in Scheme 11 [22,23].

**Figure 4:**
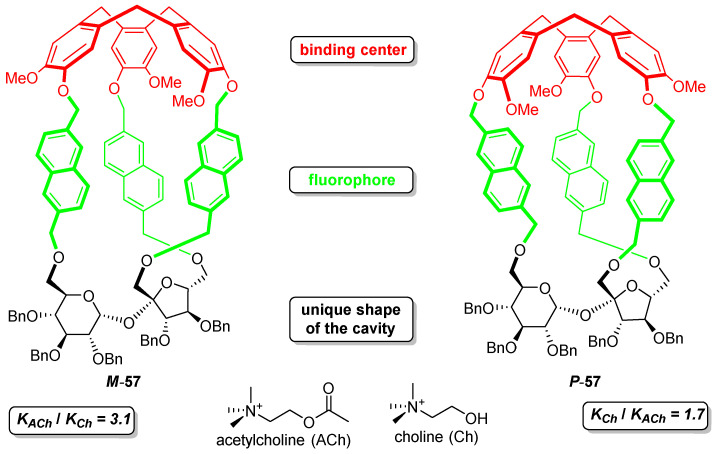
Complexing ability of two (out of four) molecular cages, **57,** against acetylcholine and choline.

Another example of such molecular cages containing CTV and sucrose moieties connected via the naphthalene linkers is shown in Figure 4 (only two out of four isomers formed are shown). These cages were found to be selective and efficient fluorogenic sensors for the detection of acetylcholine or choline. Compound ***P***-**57** has a better affinity for choline over acetylcholine, while cage ***M***-**57** exhibits a higher constant association for acetylcholine over choline [23].

**Scheme 12:**
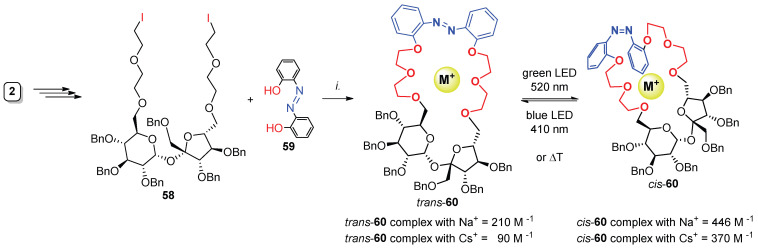
Synthesis of molecular switches with sucrose scaffold. *i.* Cs_2_CO_3_, MeCN, 82 °C, 6h, 85%.

Molecular switches built on the sucrose platform were also available, as shown in Scheme 12. Diol **2** was converted in a few simple steps into di-iodide **58,** which—upon its reaction with azobenzene **59**—gave macrocycle **60** at a high yield with the trans isomer strongly predominated over the cis one. After irradiation with green light, isomer trans-**60** underwent conversion into the cis isomer, while the irradiation of cis-**60** with blue light (or just heating) induced its conversion into the more thermodynamically stable trans isomer [24]. The complexing properties of both isomers towards achiral cations were rather low but slightly differed from each other, as shown in Scheme 12.

## 3. Complexing Properties

**Figure 5:**
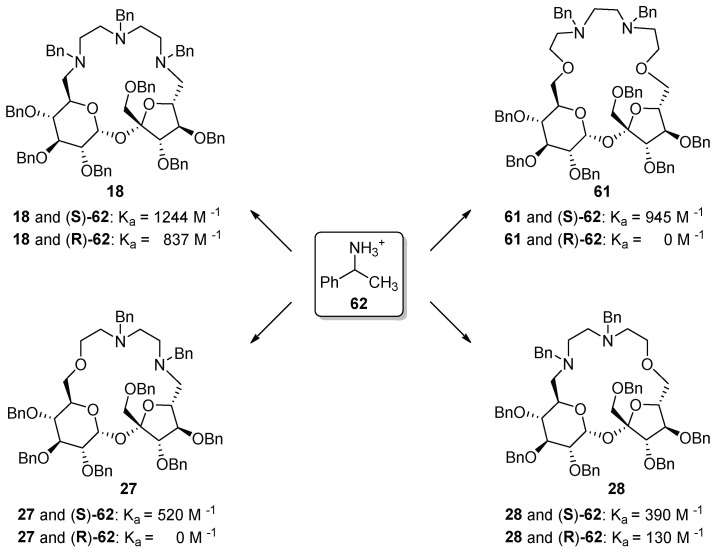
Association constants of aza-crowns **18** and **61** and unsymmetrical aza-crowns (**27**, **28**) with both enantiomers of α-phenylethylamine (**62**).

Macrocycles with a sucrose scaffold presented above (crown, aza-crown analogs, and cryptand **49**) show complexing properties towards achiral cations (mostly inorganic) or anions (ureas **45a** and **45b**). However, the application of such highly functionalized, enantiomerically pure receptors to complex achiral guest is not interesting; we performed these studies only to check if such compounds have the complexing abilities. The application of these sucrose-based hosts for the complexation of chiral guests was, however, quite promising. We proved that they are able to distinguish both enantiomers of the phenylethylamine cation; thus, the ***enantioselective*** complexation of chiral guests is possible, at least for simple model guests. The first interesting results were reported in our earlier study (from 2008) on the synthesis of sucrose-base receptors. The preferred complexation of (S)-phenylethylamine cation (**62**) was noted by receptor **18,** while receptor **61** formed a strong complex with the (S)-enantiomer only (Figure 5) [9]. This selectivity towards the (S)-**62** over the (R)-**62** was also noted for “unsymmetrical” receptors **27** and **28** (Figure 5) [12].

## 4. Conclusions

We have proved that sucrose, a very cheap raw material available in almost unlimited quantities, can be used (besides in “commercial” applications) as a building block for the synthesis of fine products with high added value. Several types of macrocyclic derivatives with sucrose scaffold are available. Some of them show interesting complexing properties and are able to differentiate between the two enantiomers of simple chiral guests.

The sucrose platform in our syntheses was fully protected with benzyl groups, which could be removed (as we proved in a few cases) under mild conditions, not affecting the very sensitive glycosidic bond. Such “free” (not protected) receptors, which are soluble in water, might find an application in studies of the complexation of chiral guests in biological environments.

Especially interesting is the field of molecular cages incorporating sucrose and the CTV unit. These two partners can be connected by appropriate linkers of different lengths, which opens a convenient way for cages with various cavities to be able to differentiate between important biologically active compounds.

Also, the molecular switches built from sucrose and azobenzene open a promising field of study. Depending on the light (green or blue), such derivatives change their configuration (from trans to cis and vice versa), thus modifying their cavity and, hence, the complexing properties towards various cations.

As shown in this short note, sucrose can also find an application in the synthesis of fine chemicals, which can be useful in many fields. Thus, this disaccharide is available in practically unlimited quantities, which is ignored in most typical organic laboratories, and can be used as a starting material for the synthesis of various types of optically pure fine chemicals.

## Data Availability

Data sharing is not applicable.

## Abbreviations

The following abbreviations are used in this manuscript:

CTV	cycloveratrylene
*tert*-BDPS-Cl	*tert*-butyldiphenyl silyl chloride
TrCl	triphenylmethyl chloride (trityl chloride)
